# Treatment of allergic rhinitis with acupoint herbal plaster: an oligonucleotide chip analysis

**DOI:** 10.1186/s12906-016-1418-0

**Published:** 2016-11-04

**Authors:** Horng-Sheng Shiue, Yun-Shien Lee, Chi-Neu Tsai, Hen-Hong Chang

**Affiliations:** 1 Chang Gung Memorial Hospital and Chang Gung University College of Medicine, Taoyuan, Taiwan; 2 Department of Biotechnology, Ming Chuan University, Taoyuan, Taiwan; 3 School of Post-Baccalaureate Chinese Medicine, and Research Center for Chinese Medicine and Acupuncture, China Medical University, Taichung, Taiwan; 4 Departments of Chinese Medicine, China Medical University Hospital, Taichung, Taiwan

**Keywords:** Allergic rhinitis, Acupoint herbal plaster, Oligonucleotide chip

## Abstract

**Background:**

Allergic rhinitis is regarded as an imbalanced Th1/Th2 cell-mediated response. The present study used microarray analysis to compare gene expression levels between allergic rhinitis patients before and after a series of acupoint herbal plaster applications.

**Methods:**

In this experimental pilot study, volunteers experiencing sneezing, runny nose, and congestion for more than 9 months in the year following initial diagnoses were included after diagnostic confirmation by otolaryngologists to exclude patients with sinusitis and nasal polyps. Patients with persistent allergic rhinitis each received four acupoint herbal plaster treatments applied using the moxibustion technique. Clinical outcomes were evaluated using the Rhinitis Quality of Life Questionnaire (RQLQ). Peripheral blood samples were analyzed using an ImmunoCAP Phadiatop test, and patients were classified as phadiatop (Ph)-positive or -negative. Microarray results were analyzed for genes that were differentially expressed between (1) Ph-positive and -negative patients treated with herbal plaster; and (2) before and after herbal plaster treatment in the Ph-positive patient group. Unsupervised and supervised methods were used for gene-expression data analysis.

**Results:**

Nineteen Ph-positive and four Ph-negative participants with persistent allergic rhinitis were included in the study. RQLQ results indicated that the 19 Ph-positive volunteers experienced improvement in six of seven categories following acupoint herbal plaster treatments, whereas the four Ph-negative participants reported improvement in only two categories. Hierarchical clustering and principle component analysis of the gene expression profiles of Ph-positive and –negative participants indicated the groups exhibited distinct physiological responses to acupoint herbal treatment. Evaluation of gene networks using MetaCore identified that the “Immune response_IL-13 signaling via JAK-STAT” and the “Inflammation_Interferon signaling” were down- and up-regulated, respectively, among Ph-positive subjects.

**Conclusions:**

In this preliminary study, we find that the IL-13 immune response via JAK-STAT signaling and interferon inflammation signaling were down- and upregulated, respectively, in the Ph-positive group. Further studies are required to verify these pathways in Ph-positive patients, and to determine the mechanism of such pathway dysregulation.

**Trial registration:**

ClinicalTrials.gov: NCT02486159. Registered 30 Jun 2015.

## Background

Many patients with allergic rhinitis have chosen complementary and alternative medicine (CAM), including traditional Chinese medicine (TCM) or acupuncture [1, 2], as they have found CAM to be more attractive and less invasive [1]. The World Health Organization (WHO) published an article examining CAM therapies for allergic rhinitis and asthma [2], which include major contributions from TCM and deserve our continued study to assess therapeutic efficacies and mechanisms. In addition to acupuncture and TCM to treat allergic rhinitis, acupoint herbal plaster applications have recently been used widely in Taiwan [3–5] and mainland China [6, 7] due to the noninvasive and easy to manipulate nature of these treatments. An herbal plaster is applied with a drug applicator using a technique akin to moxibustion, stimulating the skin at specific acupuncture points [3, 4]. Acupoint herbal plaster methods have been recommend for allergic rhinitis beginning in 2009 [8], and practitioners throughout Taiwan and China use similar approaches in the composition of herbal medicine, the herbal medicine application operating process [9] and what acupoints are used [10]. Clinical research regarding the application of acupoint therapy for allergic rhinitis has increased, and evidence-based methods have validated its efficacy and safety [7, 9–11]. However, the majority of these studies are clinical trials; therefore, the efficacy and mechanisms of acupoint herbal plaster treatment need to be validated via mechanistic, molecular methods [2, 9, 12].

We previously studied the effect of herbal plaster treatment for allergic rhinitis [13]. Ours was the first comprehensive clinical outcome assessment of acupoint herbal plaster therapy for allergic rhinitis using the Rhinoconjunctivitis and Rhinitis Quality of Life Questionnaire (RQLQ) [14]. We showed that acupoint herbal plaster for the treatment of allergic rhinitis is safe, effective, and associated with high compliance rates. Here, we aimed to perform a pilot study for acupoint herbal plaster treatment based on our previous microarray experience. Our laboratory has rich microarray experience that combines the Genomic Medicine Research Core Laboratory (GMRCL) [15], clinicians in the Department of Chinese Medicine at Chang Gung Memorial Hospital, and bioinformatics specialists. We performed chip analysis before and after acupuncture treatment in allergic rhinitis patients [16, 17]. We used cDNA microarray and oligonucleotide microarray analyses to investigate the influence of acupuncture on RNA expression profiles using blood samples from patients with allergic rhinitis. We used the RQLQ and statistical analysis to assess clinical outcomes [14]. The results of our microarray analysis were associated with the RQLQ to obtain our final conclusions.

Following exposure to allergens, allergic rhinitis patients exhibit immunoglobulin E (IgE), mast cell, and T helper (Th)2 lymphocyte immune responses related to (1) sensitization and memory, (2) the early phase, and (3) the late phase [18, 19]. The early phase can induce sneezing, nasal itching, runny and congested nasal passages, and other symptoms. The late phase contributes to patient fatigue, malaise, irritability, and other symptoms. Allergic rhinitis is regarded as an imbalanced Th1/Th2 cell-mediated response [20, 21]. Th1 cells primarily secrete IL-2, IFNγ, IL-3, and GM-CSF; whereas Th2 cells secrete IL-3, IL-4, IL-5, IL-10, IL-13, and GM-CSF [22]. Dominant Th2 cytokines can enhance allergen-specific IgE, which plays an important role in allergic inflammation [18, 20]. Studies using DNA microarray have indicated an imbalance in the T-helper cell-mediated immune system in patients with allergic rhinitis [23, 24]. Genes encoding chemokines and their receptors were elevated in this analysis; these genes play important roles in the Th2 response [24, 25].

According to our previous study, peripheral blood samples collected from allergic rhinitis patients before and after acupuncture treatment and analyzed by cDNA microarray analysis indicated an improvement in the counterbalance between pro-inflammatory cytokines derived from Th1 cells and anti-inflammatory cytokines derived from Th2 cells [16]. Nasal allergic reactions in patients with allergic rhinitis were inhibited by Th1 cells and were not promoted by Th2 cells following acupuncture treatment [16]. Although strengthening the Th1 response is regarded as a novel therapeutic target for allergic rhinitis, it has not yet been applied in clinical practice [19, 21]. We have published that acupuncture treatment may be another way to restructure Th1 and Th2 responses in patients with allergic rhinitis [16]. ImmunoCAP Phadiatop is a blood test widely used by ENT specialists in Taiwan to detect serum allergen-specific IgE antibodies [26, 27]. Among normal controls and atopic patients, the frequency of Ph-positive patients was 1 of 47 and 49 of 53, respectively [26]. In our previous study [17], Th1 and Th2 cells were suppressed after acupuncture treatment with group differences between Phadiatop (Ph)-positive and Ph-negative patients regarding gene expression characteristics and physiological responses. Studies have shown that the reduction in allergic inflammation and the restored Th1/Th2 (and Treg/Th2) equilibrium following acupuncture are sustained [17].

In this pilot study, we examined changes in gene expression associated with acupoint herbal plaster for allergic rhinitis. Using microarray, we compared gene expression levels in allergic rhinitis patients before and after a series of acupoint herbal plaster applications. This study applies EBM and supports the use of acupoint herbal therapy to treat allergic rhinitis.

## Methods

### Acupoint herbal plaster treatment

This pilot study was designed using an intervention model with single group assignment. Allergic rhinitis patients were included after their diagnoses were confirmed, and were treated with four applications of herbal plaster. The clinical portion of this study was conducted at the Department of Acupuncture and Moxibustion, Center for Traditional Chinese Medicine, Chang Gung Memorial Hospital from October 2009 to March 2010. Patients (age, 18–45 y) were eligible who met the following criteria: (1) exhibited sneezing, runny nose, and congestion for more than 9 months of the year [18]; (2) did not take medication in the previous month; and (3) provided written consent to enter a Chang Gung Memorial Hospital Institutional Review Board (IRB)-approved human trial. Patient diagnoses were confirmed by the following clinical and biochemical tests, which were performed by otolaryngologists: (1) physical examination; (2) anterior rhinoscopy; (3) ImmunoCAP Phadiatop (InVitroSight, Phadia AB, Uppsala, Sweden), determination of specified serum IgE antibodies to detect inhalant allergens [26, 27]. Patients were included in the trial after their initial diagnoses were confirmed [18, 28]. Patients with sinusitis or nasal polyps, or those who were unwilling or unable to complete the full course of treatment were excluded from the trial. All included patients were diagnosed with allergic rhinitis that was consistent with persistent allergic rhinitis according to ARIA’s new classification system. The ARIA system includes the following rhinitis symptoms and quality of life variables: duration, which includes intermittent or persistent allergic rhinitis; and nasal allergy symptoms, which must occur more than 4 days per week for 4 months per year to qualify as persistent allergic rhinitis [29, 30].

In total, 23 study patients received acupoint herbal plaster applications every 7–10 days over a 4-week period for a total of 4 applications. The herbal plaster consisted of mustard seed, fumarate, asarum, angelica, cinnamon, and ginger at a ratio of 3:3:2:2:0.5:4, respectively. The treatment was prepared by dissolving the ginger in water and adding the powder to form a plaster. Mixtures were formed into cakes of approximately 1.5 × 1.5 × 0.5 cm^3^ [13] and were held in position using plastic sheets. The following nine acupoints were selected: Dazhui (GV14), Feishu (BL13, both sides), Gaohuang (BL43, both sides), Shenshu (BL23, both sides), and Pishu (BL20, both sides). Each patching time lasted 1–3 h, depending on the patient’s tolerance. When drug cakes were removed, patients typically exhibited local skin redness and experienced slight burning sensations. Subsequent water exposure, including bathing, was avoided for 1–2 h following treatment to prevent skin aggravation. Patient drug tolerance varies, and adhering the cake for too long occasionally led to blisters. Blisters resulting from this treatment were coated with povidone iodine syrup and were protected with sterile gauze bandages.

### Outcome evaluation

Clinical symptoms were indexed as follows: (1) assess symptoms before the first acupoint herbal plaster application, (2) determine rhinoconjunctivitis and rhinitis symptoms at the third and fourth acupoint herbal plaster applications. Clinical outcomes were evaluated using the RQLQ, which has been proven to be effective [14, 31] and includes 28 questions in 7 categories. The RQLQ was designed to measure the impact of rhinitis on quality of life. It considers that allergic rhinitis patients often are troubled by nasal symptoms, eye symptoms, sleep problems, emotional problems, social issues, and other symptoms [14, 29].

### ImmunoCAP Phadiatop blood test

Prior to treatment at Chang Gung Memorial Hospital, all 23 allergic rhinitis patients were assessed by clinical pathologists using the ImmunoCAP Phadiatop blood test. Patients were evaluated for the presence of IgE antibodies against the following allergens: *Dermatophagoides pteronyssinus*, cat dander, dog dander, the German cockroach, and Moulds. Detection of IgE antibodies exceeding 0.35 kUA/L indicated a positive result.

### RNA extraction and microarray

Patient peripheral blood samples were obtained in 5-ml volumes at the following 6 times (T0–T5) during the study: (1) before (T0) and 24 h after the first (T1) acupoint herbal plaster application; (2) before (T2) and 24 h after the third (T3) acupoint herbal plaster application; and (3) before the fourth (T4) and 24 h after the fourth (T5) acupoint herbal plaster application.

From each 5-ml blood sample, 2.5-ml aliquots were analyzed by the Clinical Pathology Department of Chang Gung Memorial Hospital for the following: complete blood count/differential count (CBC/DC):total white blood count; differential counts for neutrophils, lymphocytes, monocytes, eosinophils, and basophils; red blood cell count; platelet count; hemoglobin, hematocrit, and erythrocyte indices (mean corpuscular volume, mean corpuscular hemoglobin, mean corpuscular hemoglobin concentration, and red cell distribution width [RDW]). Total serum IgE levels were tracked before the first acupoint herbal plaster application and 24 h after the fourth acupoint herbal plaster application.

The remaining 2.5-ml blood samples were stored at room temperature in PAXgene Blood RNA collection tubes (Qiagen, Valencia, CA, USA), containing an RNA stabilizer. RNA was extracted from blood samples using the PAXgene Blood RNA System (Qiagen), according to the manufacturer’s recommendations, and samples were stored at −80 °C. RNA samples then were isolated using an RNeasy MinElute kit (Qiagen), and RNA quality and quantity were analyzed using a Bioanalyzer 2100 (Agilent Technologies, Santa Clara, CA, USA).

Owing to the IRB’s limitation that no more than 5-ml peripheral blood could be collected from each study volunteer, we were unable to obtain sufficient RNA quantities to analyze individual participants. Therefore, we applied pre-amplification pooled mRNA samples to a single microarray chip, a method that has been used frequently in microarray analysis [23]. Although pooling could potentially confound signals by mixing cell populations and individuals, it avoids variation within individuals [32]. Because a microarray using pooled RNA only identifies genes that change dramatically, this approach highlights the most differentially expressed signaling pathways between diseased and control individuals [25]. In our study, equal quantities of mRNA were pooled from individuals with similar clinical diagnosis and IgE levels, thereby increasing RNA homogeneity. Each pooled sample corresponded to the blood RNA from 2 to 3 patients. Samples were analyzed using a GeneChip Human Genome U133 Plus2 array (Affymetrix, Santa Clara, CA, USA) containing approximately 54,675 probes. Samples from the 23 patients were divided into 7 pooled groups for each of the 6 blood collection time points and were applied to 42 chips.

### Statistical analysis

Changes in RQLQ and IgE were compared to the first time point (T0; before first herbal plaster) via a paired Student’s *t*-test and a Mann Whitney *U*-test, respectively.

### Microarray data analysis

Unsupervised (hierarchical clustering and principal component analysis) and supervised (Student’s *t*-test) methods have traditionally been used to analyze gene-expression data [33]. In this study, data were analyzed by hierarchical clustering using Cluster and TreeView software [34] with the following parameters: (1) standard deviation > 0.4 as the filtering cutoff point (1852 genes with marked changes selected among 35 arrays); (2) mean-centered genes and normalized genes; and (3) cluster analysis conducted using uncentered correlation of arrays. Cluster and TreeView programs were downloaded from http://bonsai.hgc.jp/~mdehoon/software/cluster. The Student’s *t*-test, Mann–Whitney *U*-test and PCA were performed using MATLAB version 7.4 and Statistics Toolbox version 3.1 (The MathWorks, Boston, MA, USA). A volcano plot was constructed using MATLAB to identify changes in replicate microarray data [35]. Specifically, the negative log of the p value (−log10[p value]) was plotted on the y-axis, and the log2 ratio of the fold change was plotted on the x-axis.

We evaluated genes that were differentially expressed following acupoint herbal plaster applications (T1, T2, T3, T4, T5, are compared with T0). Changes in specific gene expression before and after treatment could suggest potential immune mechanisms associated with acupoint herbal plaster application. RQLQ results were compared with gene expression differences in the final analysis.

### Network visualization and analysis

The MetaCore analytical suite (GeneGo, St. Joseph, MI, USA) was used to compare differences in gene expression networks [36–39]. MetaCore evaluates systems biology and drug development at the computational level, enabling analyses of human protein–protein interactions and mechanisms using the database. This suite contributes to analyses of regulatory networks and signaling pathway gene groups. To perform a network analysis of gene groups, MetaCore can work from an input list of genes and can randomly assign genes to different nodes to assess the probability of an interacting network [37]. In this study, the list of genes represented on the Affymetrix Human U133 Plus2 array was used as a base gene list to calculate p values using MetaCore procedures. MetaCore uses a hypergeometric model to determine significance [38, 39].

## Results

### Clinical outcomes of acupoint herbal plaster treatment

An otolaryngologist screened 23 study participants with allergic rhinitis, and the GMRCL conducted oligonucleotide chip experiments. Each participant’s diagnosis of perennial allergic rhinitis also was confirmed using anterior nasal endoscopy. Based on the results of an ImmunoCAP Phadiatop blood test of allergen-specific IgE, the 23 volunteers were classified as either Ph-positive (19 participants) or Ph-negative (4 participants) (Table 1). Assessments of clinical symptoms and IgE indices were performed before the first, third, and after the fourth acupoint herbal plaster application. The RQLQ was used to survey the patients, and the results were statistically analyzed for clinical symptoms [14] (Tables 2 and 3).

**Table 1:** Comparison of baseline characteristics between Ph-positive and Ph-negative patients before treatment

Variables	Ph-positive *N* = 19	SD	Ph-negative *N* = 4	SD	*p* Value
	Mean		Mean		
Gender					
Male	10		3		
Female	9		1		0.60^
Age	32.11	5.37	35	3.37	0.22
Duration of allergic rhinitis					
≥ 10 years	14		3		
< 10 years	5		1		0.96^
Activity	3.12	1.39	3.08	1.32	0.66
Sleep	1.65	1.09	1.58	0,92	0.64
Non-hay fever symptoms	2.39	1.14	2.25	1.08	0.58
Practical problems	2.84	1.42	2.33	1.61	0.38
Nasal symptoms	2.78	1.17	2.94	1.43	0.98
Eye symptoms	2.37	1.41	1.75	1.14	0.24
Emotional symptoms	2.08	1.15	1.38	0.92	0.15
Overall score	2.46	1.02	2.19	0.96	0.40
IgE (Baseline)	302.12	78.75	21.25	7.70	0.002**
IgE (Follow-up)	333.61	86.01	25.10*	10.44	0.005**

*SD* Standard Deviation

*Note*: *p < 0:05, **p < 0:01 (Mann–Whitney *U* test)

^Fisher’s exact test

**Table 2:** Changes in RQLQ results following the third and fourth herbal plaster (hp) treatments in Ph-positive patients

Area of RQLQ	Baseline score	After 3^rd^ hp score	*P* value (3^rd^ hp vs. baseline)	After 4^th^ hp score	*P* value (4^th^ hp vs. baseline)
Activity	3.12	2.56	0.1322	2.09	0.0002**
Sleep	1.65	1.58	0.8488	1.35	0.0804
Non-hay fever symptoms	2.39	2.02	0.1465	1.64	0.0012**
Practical problems	2.84	2.39	0.1549	2.05	0.0018**
Nasal symptoms	2.78	2.30	0.1006	1.92	0.0000**
Eye symptoms	2.37	1.57	0.0330*	1.29	0.0066**
Emotional symptoms	2.08	1.62	0.0634	1.33	0.0010**
Overall score	2.46	2.00	0.0635	1.67	0.0000**

Paired Student’s *t*-test; *n* = 19, *p < 0.05 **p < 0.01

**Table 3:** Changes in RQLQ results following the third and fourth herbal plaster (hp) treatments in Ph-negative patients

Area of RQLQ	Baseline score	After 3^rd^hp score	*P* value (3^rd^ hp vs. baseline)	After 4^th^hp score	*P* value (4^th^ hp vs. baseline)
Activity	3.08	1.58	0.0577	1.33	0.0800
Sleep	1.58	1.58	1.0000	1.17	0.3677
Non-hay fever symptoms	2.25	1.79	0.3477	1.21	0.0564
Practical problems	2.33	1.67	0.3994	1.33	0.1135
Nasal symptoms	2.94	2.06	0.1881	1.31	0.0065**
Eye symptoms	1.75	1.19	0.4338	1.19	0.4594
Emotional symptoms	1.38	1.06	0.5551	0.69	0.0486*
Overall score	2.19	1.56	0.1940	1.18	0.0371*

Paired Student’s *t*-test; *n* = 4, *p < 0.05 **p < 0.01

In the Ph-positive group, the RQLQ results were compared before the first and after the fourth acupoint herbal plaster treatment. We identified significant improvements in six of the seven domains (activity, non-hay fever symptoms, eye symptoms, practical problems, nasal symptoms, and emotional symptoms) examined by the RQLQ (Tables 2 and 3). In the Ph-negative group, only two categories (nasal symptoms, emotional symptoms) appeared to improve following acupoint treatment. These results suggest that acupoint herbal plaster applications evoke distinct physiological responses in these two patient groups. These findings are consistent with our previous studies regarding acupuncture treatment for allergic rhinitis [16, 17].

Total serum IgE values were compared before the first and after the fourth acupoint herbal plaster application (Tables 4 and 5). Following the course of herbal plaster treatments, total IgE levels were unchanged in both the Ph-positive and -negative groups (Tables 4 and 5). This is consistent with previous short-term studies by our laboratory [16, 17] and others [40], which found that total serum IgE levels in allergic rhinitis patients treated with TCM did not change.

**Table 4:** Changes in total IgE levels following the fourth herbal plaster (hp) treatment in Ph-positive patients

	No.	Baseline	Follow-up	*P* value^
		Mean ± SD	Mean ± SD	
IgE	19	302.12 ± 78.75	333.61 ± 86.01	0.085

*SD* Standard Deviation

^Mann–Whitney *U*-test

**Table 5:** Changes in patient total IgE levels following the fourth herbal plaster (hp) treatment in Ph-negative patients

	No.	Baseline	Follow-up	*P* value^
		Mean ± SD	Mean ± SD	
IgE	4	21.25 ± 7.70	25.10 ± 10.44	0.63

*SD* Standard Deviation

^Mann–Whitney *U*-test

### Ph-positive and Ph-negative allergic rhinitis patients exhibit distinct gene expression profiles following acupoint herbal plaster treatment

Since Ph-positive and Ph-negative groups exhibited different clinical outcomes, we explored the gene expression profiles of these two patient groups following acupoint herbal plaster treatment. Total RNA was extracted from peripheral blood samples at each of the 6 time points analyzed (23 patients, 138 RNA samples total). Because of insufficient blood RNA quantities (1–2 μg/subject), we pooled sets of 2–3 RNA samples from subjects with similar clinical indices, resulting in seven pooled RNA samples for each of the six time points. The 42 pooled RNA samples were applied to GeneChip Human Genome U133 Plus 2.0 arrays. Patient and sample information are detailed in Table 6.

**Table 6:** Pooling strategy for RNA samples. The first number in each cell indicates the group type, and the second indicates the time point (T0–T5 correspond to 1–6, respectively). A total of 42 chips were used. M, microarray chip

	Before 1^st^ herbal plaster (hp) (T0)	After 1^st^ hp 24 h (T1)	Before 3^rd^hp (T2)	After 3^rd^ hp 24 h (T3)	Before 4^th^hp (T4)	After 4^th^ hp 24 h (T5)
Ph(+)	M1-1	M1-2	M1-3	M1-4	M1-5	M1-6
Ph(+)	M2-1	M2-2	M2-3	M2-4	M2-5	M2-6
Ph(+)	M3-1	M3-2	M3-3	M3-4	M3-5	M3-6
Ph(+)	M4-1	M4-2	M4-3	M4-4	M4-5	M4-6
Ph(+)	M5-1	M5-2	M5-3	M5-4	M5-5	M5-6
Ph(+)	M6-1	M6-2	M6-3	M6-4	M6-5	M6-6
Ph(−)	M7-1	M7-2	M7-3	M7-4	M7-5	M7-6

**Fig. 1:**
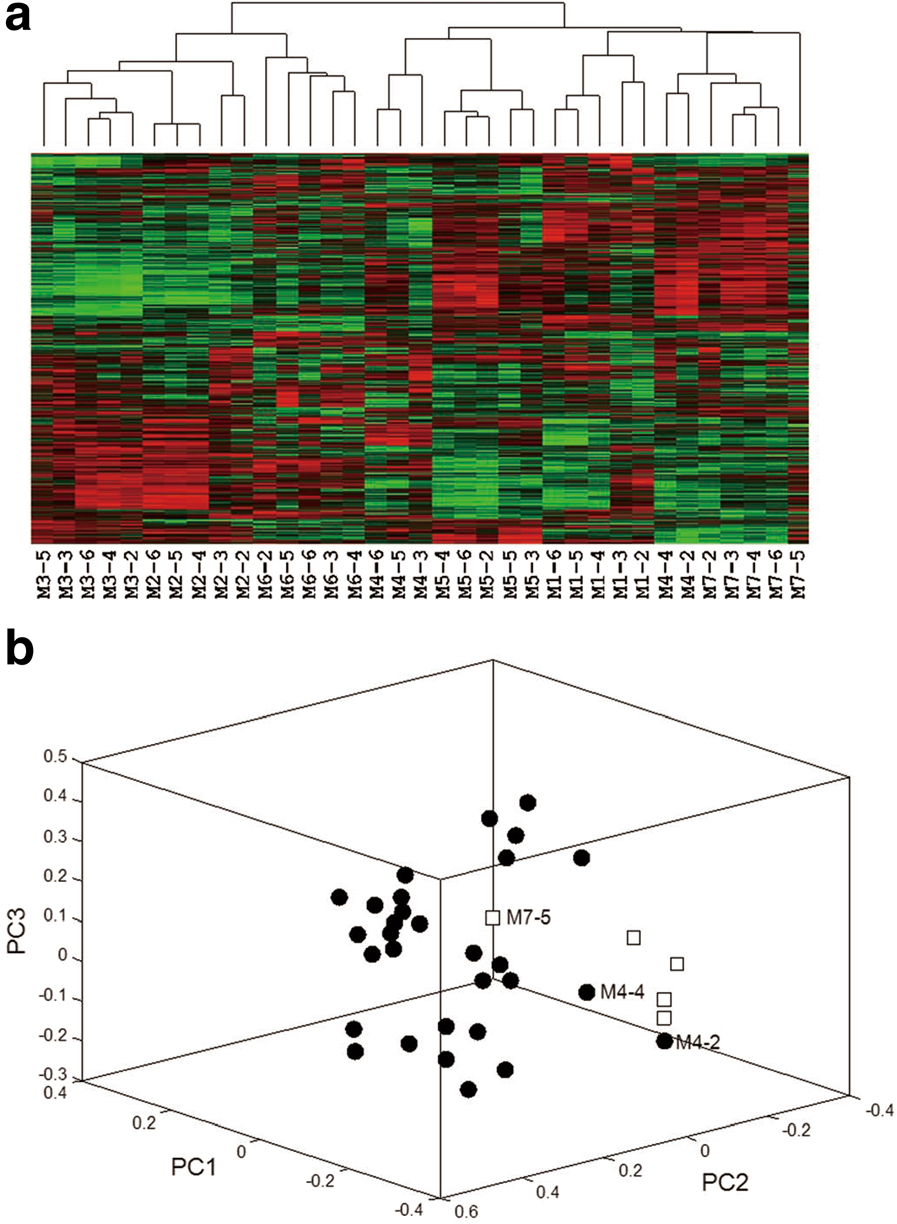
Distinct gene expression profiles in Ph-positive and -negative patient groups as determined by **a** hierarchical clustering and **b** principle component analysis. **a** Each column represents a chip, and each row represents a specific gene. The gene expression level at each treatment point was subtracted from the first time point (T0). The color map uses red and green for high and low expression values, respectively. Black corresponds to genes exhibiting non-significant variation. **b** Three-dimensional plot of the first three principal components by the matrix containing 80 % of the information. Ph-positive and -negative patients are indicated as closed squares and open circles, respectively

To estimate the effects of acupoint herbal plaster treatment, the gene expression level at each treatment point was subtracted from the first time point (T0; before herbal plaster treatment). After filtering the low-intensity non-significant genes (standard deviation < 0.4), 1852 genes remained for analysis with non-supervised hierarchical clustering methods. We identified distinct gene expression profiles in Ph-positive and -negative patients using a hierarchical approach (Fig. 1a). We further analyzed the correlation matrix for all 35 samples using a PCA [41]. The three-dimensional plot of the first three principal components by the matrix containing 80 % of the information is shown in Fig. 1b. This analysis indicated that the Ph-positive and -negative groups were distinct in their responses to acupoint herbal plaster treatment. Because the hierarchical clustering and PCA suggested that the M4-2 and M4-4 samples were outliers in the Ph-positive group, these samples were excluded from further analysis.

**Fig. 2:**
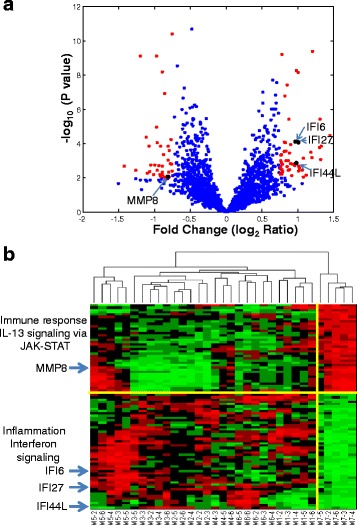
**a** Volcano plot of the 1852 filtered genes and **b** functional and clustering analyses of the differentially expressed genes between Ph-positive and -negative groups. **a** In the volcano plot, the -log10(P value) is plotted on the y-axis, and the log2 ratio of the fold change is plotted on the x-axis. In total, 89 genes (red points) that exhibited fold-changes exceeding 2^0.75^ = 1.682 between Ph-positive and -negative groups were selected from the 1852 filtered genes (*p* < 0.01, Student’s *t*-test). **b** The 89 differentially expressed genes were analyzed with MetaCore software, and “Immune response_IL-13 signaling via JAK-STAT” and “Inflammation_Interferon signaling” pathways were found to correspond to up- and down-regulated genes, respectively, in Ph-positive patients. The genes involved in pathway are indicated with arrows

Since, the clinical outcomes (RQLQ) after treatment in the Ph-positive and -negative groups differed, we explored the gene expression profiles for these two groups in response to acupoint herbal plaster application. We used a volcano plot to obtain an overview of the 1852 filtered genes (Fig. 2a), and we selected 89 genes that exhibited fold-changes exceeding 2^0.75^ = 1.682 (*p* < 0.01, Student’s *t*-test) between Ph-positive and -negative participants (Fig. 2b and Table 7). These genes were examined using MetaCore software (http://lsresearch.thomsonreuters.com/pages/solutions/1/metacore) for reaction pathway analysis, and the pathways “Immune response_IL-13 signaling via JAK-STAT (Janus kinase and signal transducers and activators of transcription)” and “Inflammation_Interferon signaling” were identified to correspond to the down- and up-regulated genes, respectively, in the Ph-positive group (Fig. 2b and Table 8).

**Table 7:** The 89 genes that were differentially expressed between Ph-positive and Ph-negative patients with allergic rhinitis following treatment with acupoint herbal paste

ID	Gene Symbol	Gene Title	Fold change^a^	*P* value
1552288_at	CILP2	cartilage intermediate layer protein 2	1.45	3.4E-05
1556590_s_at	NA	NA	1.32	1.4E-04
1557195_at	NA	NA	1.31	8.8E-04
1557761_s_at	LOC400794	hypothetical LOC400794	1.31	3.9E-06
1562216_at	NA	NA	1.30	1.7E-04
1565913_at	NA	NA	1.21	4.2E-10
1566134_at	CARHSP1	Calcium regulated heat stable protein 1, 24 kDa	1.20	6.5E-04
1566964_at	NA	NA	1.18	3.4E-04
1567240_x_at	OR2L2	olfactory receptor, family 2, subfamily L, member 2	1.11	7.1E-03
1569482_at	NA	NA	1.08	8.8E-03
200038_s_at	RPL17	ribosomal protein L17	1.08	7.4E-04
200082_s_at	RPS7	ribosomal protein S7	1.06	3.7E-03
200705_s_at	EEF1B2	eukaryotic translation elongation factor 1 beta 2	1.02	2.6E-03
200986_at	SERPING1	serpin peptidase inhibitor, clade G (C1 inhibitor), member 1	1.02	2.0E-03
201699_at	PSMC6	proteasome (prosome, macropain) 26S subunit, ATPase, 6	1.01	4.0E-03
202086_at	MX1	myxovirus (influenza virus) resistance 1, interferon-inducible protein p78 (mous	1.01	7.2E-09
202411_at	IFI27	interferon, alpha-inducible protein 27	1.00	7.8E-05
202635_s_at	POLR2K	polymerase (RNA) II (DNA directed) polypeptide K, 7.0 kDa	0.99	1.4E-03
204286_s_at	PMAIP1	phorbol-12-myristate-13-acetate-induced protein 1	0.98	5.5E-09
204415_at	IFI6	interferon, alpha-inducible protein 6	0.97	7.6E-05
204439_at	IFI44L	interferon-induced protein 44-like	0.96	2.0E-03
204732_s_at	TRIM23	tripartite motif-containing 23	0.93	1.7E-03
205849_s_at	UQCRB	ubiquinol-cytochrome c reductase binding protein	0.91	7.7E-03
205914_s_at	GRIN1	glutamate receptor, ionotropic, N-methyl D-aspartate 1	0.90	1.5E-03
206584_at	LY96	lymphocyte antigen 96	0.90	1.2E-04
207723_s_at	KLRC3	killer cell lectin-like receptor subfamily C, member 3	0.88	3.6E-04
208792_s_at	CLU	clusterin	0.88	3.5E-03
209160_at	AKR1C3	aldo-keto reductase family 1, member C3 (3-alpha hydroxysteroid dehydrogenase, t	0.88	3.9E-06
209651_at	TGFB1I1	transforming growth factor beta 1 induced transcript 1	0.86	1.0E-03
209732_at	CLEC2B	C-type lectin domain family 2, member B	0.86	3.7E-03
209743_s_at	ITCH	itchy E3 ubiquitin protein ligase homolog (mouse)	0.85	2.3E-03
209795_at	CD69	CD69 molecule	0.85	3.9E-08
210103_s_at	FOXA2	forkhead box A2	0.84	9.9E-04
210432_s_at	SCN3A	sodium channel, voltage-gated, type III, alpha subunit	0.83	2.8E-04
210548_at	CCL23	chemokine (C-C motif) ligand 23	0.83	8.5E-05
210639_s_at	ATG5	ATG5 autophagy related 5 homolog (S. cerevisiae)	0.82	1.7E-07
210873_x_at	APOBEC3A	apolipoprotein B mRNA editing enzyme, catalytic polypeptide-like 3A	0.82	2.7E-04
211968_s_at	HSP90AA1	heat shock protein 90 kDa alpha (cytosolic), class A member 1	0.81	8.0E-05
212270_x_at	RPL17	ribosomal protein L17	0.81	2.9E-04
212537_x_at	RPL17	ribosomal protein L17	0.78	1.6E-03
213226_at	CCNA2	cyclin A2	0.78	4.2E-03
214070_s_at	ATP10B	ATPase, class V, type 10B	0.78	3.3E-03
215101_s_at	CXCL5	chemokine (C-X-C motif) ligand 5	0.78	6.0E-03
215394_at	PIK3C3	phosphoinositide-3-kinase, class 3	0.77	6.3E-10
215646_s_at	VCAN	versican	0.77	4.3E-04
216412_x_at	LOC100290557	similar to hCG91935	0.77	1.3E-03
216834_at	RGS1	regulator of G-protein signaling 1	0.76	2.3E-03
217915_s_at	RSL24D1	ribosomal L24 domain containing 1	0.76	9.8E-04
219519_s_at	SIGLEC1	sialic acid binding Ig-like lectin 1, sialoadhesin	0.76	4.7E-04
219551_at	EAF2	ELL associated factor 2	0.76	3.1E-03
220141_at	C11orf63	chromosome 11 open reading frame 63	0.75	5.8E-03
220184_at	NANOG	Nanog homeobox	−0.75	3.9E-03
220646_s_at	KLRF1	killer cell lectin-like receptor subfamily F, member 1	−0.75	3.9E-11
220827_at	NA	NA	−0.76	1.6E-03
222229_x_at	RPL26	ribosomal protein L26	−0.77	5.9E-05
222465_at	RSL24D1	ribosomal L24 domain containing 1	−0.78	1.5E-03
223963_s_at	IGF2BP2	insulin-like growth factor 2 mRNA binding protein 2	−0.79	2.5E-04
224293_at	TTTY10	testis-specific transcript, Y-linked 10 (non-protein coding)	−0.79	8.6E-03
225541_at	RPL22L1	ribosomal protein L22-like 1	−0.80	4.7E-03
226344_at	ZMAT1	zinc finger, matrin type 1	−0.81	1.4E-04
227454_at	TAOK1	TAO kinase 1	−0.81	9.4E-04
227766_at	LIG4	ligase IV, DNA, ATP-dependent	−0.82	9.9E-03
228174_at	SCAI	suppressor of cancer cell invasion	−0.83	8.5E-03
228439_at	BATF2	basic leucine zipper transcription factor, ATF-like 2	−0.83	9.6E-03
228970_at	ZBTB8OS	zinc finger and BTB domain containing 8 opposite strand	−0.86	1.1E-07
229431_at	RFXAP	regulatory factor X-associated protein	−0.86	8.8E-03
229437_at	MIR155HG	MIR155 host gene (non-protein coding)	−0.87	4.5E-04
229893_at	FRMD3	FERM domain containing 3	−0.89	7.0E-03
229910_at	SHE	Src homology 2 domain containing E	−0.89	2.0E-03
230153_at	NEK9	NIMA (never in mitosis gene a)- related kinase 9	−0.89	6.2E-09
231014_at	TRIM50	tripartite motif-containing 50	−0.89	4.8E-03
231038_s_at	NA	NA	−0.92	8.1E-03
231484_at	NA	NA	−0.92	1.5E-04
231688_at	MMP8	matrix metallopeptidase 8 (neutrophil collagenase)	−0.93	6.4E-03
231975_s_at	MIER3	mesoderm induction early response 1, family member 3	−0.94	1.9E-03
233015_at	MBNL1	muscleblind-like (Drosophila)	−0.96	3.7E-03
235762_at	TAS2R14	taste receptor, type 2, member 14	−0.97	8.7E-05
236495_at	NA	NA	−0.97	8.1E-10
236666_s_at	LRRC10B	leucine rich repeat containing 10B	−0.98	1.1E-05
237689_at	SARS	Seryl-tRNA synthetase	−1.00	1.8E-03
238174_at	NA	NA	−1.01	6.3E-03
238918_at	NA	NA	−1.06	1.7E-03
239655_at	NA	NA	−1.07	4.2E-03
239819_at	NA	NA	−1.08	1.4E-04
240145_at	NA	NA	−1.10	5.3E-03
240262_at	NA	NA	−1.11	4.2E-05
240652_at	NA	NA	−1.20	8.0E-10
240866_at	NA	NA	−1.26	3.8E-03
242625_at	RSAD2	radical S-adenosyl methionine domain containing 2	−1.43	2.0E-03

*NA* Not Available

^a^fold change (Log_2_ ratio)

**Table 8:** Metacore process map for the 89 genes that were differentially expressed between Ph-positive and Ph-negative patients with allergic rhinitis following acupoint herbal paste treatment

Process map of down-regulated genes in Ph(+)			
Maps	*P* value	Filter Genes^a^	Map genes^b^
DNA damage_NHEJ mechanisms of DSBs repair	1.4E-02	1 (LIG4)	19
Neurophysiological process_Bitter taste signaling	2.0E-02	1 (TAS2R14)	28
Apoptosis and survival_Granzyme A signaling	2.1E-02	1 (LIG4)	30
Cell cycle_Role of Nek in cell cycle regulation	2.3E-02	1 (NEK9)	32
Development_Role of Activin A in cell differentiation and proliferation	2.9E-02	1 (NANOG)	40
Immune response_IL-13 signaling via JAK-STAT	3.1E-02	1 (MMP8)	44
Process map of up-regulated genes in Ph(+)			
Maps	*P* value	Filter Genes^a^	Map genes^b^
Inflammation_Interferon signaling	1.1E-02	3 (IFI6,IFI27, MX1)	110
Autophagy_Autophagy	2.3E-02	2 (PIK3C3,ATG5)	55
Cell cycle_S phase	2.6E-02	2 (HSP90AA1, CCNA2)	149

^a^Number of filter genes in the map

^b^Number of genes in the map

### Differentially expressed genes after acupoint herbal plaster treatment in Ph-positive patients

**Fig. 3:**
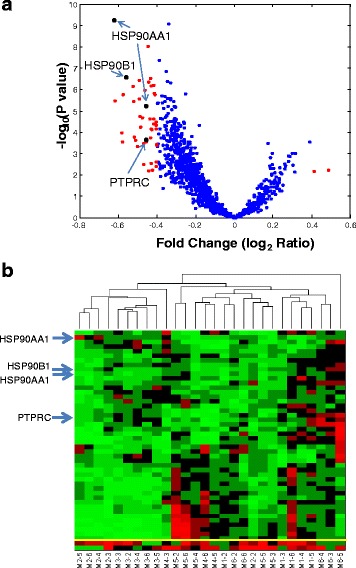
**a** Volcano plot of the gene expression profiles in the Ph-positive group and **b** differentially expressed genes (compared with T0) subjected to hierarchical clustering analysis. **a** In the volcano plot, the -log10(P value) is plotted on the y-axis, and the log2 ratio of the fold change is plotted on the x-axis. Forty-seven genes (red points) that exhibited fold-changes of 2^0.4^ = 1.320 (*p* < 0.01) were selected from the 1852 filtered genes. **b** In the cluster plot, each column represents a chip, and each row represents a specific gene. Most genes were down-regulated (45/47 genes) among the differentially expressed genes after herbal plaster treatment. The genes involved in pathway are indicated with arrows

The RQLQ indicated that the clinical efficacy of herbal plaster treatment was different between Ph-positive patients and Ph-negative patients. Then we evaluated genes that were differentially expressed following acupoint herbal plaster applications (T1, T2, T3, T4, T5, are compared with T0) in Ph-positive patients. Since the differentially expresse in Ph-positive group is less than Ph-positive group compared with Ph-negative group. We selected 47 genes that exhibited *p* < 0.01 (via Student’s *t*-test) and fold changes (vs. T0) of 2^0.4^ = 1.320 (Fig. 3 and Table 9). Globally, most genes were down-regulated (45/47) after herbal plaster treatment. This result was consistent with our previous report that most genes were down-regulated after acupuncture treatment in Ph-positive allergic rhinitis patients [17].

**Table 9:** The 47 genes that were differentially expressed as compared to the first time point (T0; before herbal plaster treatment in the Ph-positive group)

ID	Gene Symbol	Gene Title	Fold change^a^	*P* value
211969_at	HSP90AA1	heat shock protein 90 kDa alpha (cytosolic), class A member 1	−0.62	5.4E-10
224567_x_at	MALAT1	Metastasis associated lung adenocarcinoma transcript 1 (non-protein coding)	−0.62	3.3E-06
226675_s_at	MALAT1	Metastasis associated lung adenocarcinoma transcript 1 (non-protein coding)	−0.58	1.0E-04
216563_at	ANKRD12	Ankyrin repeat domain 12	−0.58	2.8E-04
222465_at	RSL24D1	ribosomal L24 domain containing 1	−0.58	1.6E-06
204732_s_at	TRIM23	tripartite motif-containing 23	−0.56	2.8E-07
201304_at	NDUFA5	NADH dehydrogenase (ubiquinone) 1 alpha subcomplex, 5, 13 kDa	−0.52	6.9E-07
203491_s_at	CEP57	centrosomal protein 57 kDa	−0.52	3.5E-06
235643_at	SAMD9L	sterile alpha motif domain containing 9-like	−0.52	1.7E-04
209662_at	CETN3	centrin, EF-hand protein, 3 (CDC31 homolog, yeast)	−0.51	2.3E-04
212417_at	SCAMP1	secretory carrier membrane protein 1	−0.50	4.5E-04
217915_s_at	RSL24D1	ribosomal L24 domain containing 1	−0.49	2.6E-06
200598_s_at	HSP90B1	heat shock protein 90 kDa beta (Grp94), member 1	−0.49	3.6E-07
242429_at	ZNF567	zinc finger protein 567	−0.49	1.8E-05
232958_at	NA	NA	−0.48	5.6E-05
222326_at	NA	NA	−0.48	2.0E-05
200026_at	RPL34	ribosomal protein L34	−0.47	3.1E-03
221765_at	UGCG	UDP-glucose ceramide glucosyltransferase	−0.47	3.1E-04
212794_s_at	KIAA1033	KIAA1033	−0.46	1.0E-06
200099_s_at	RPS3A	ribosomal protein S3A	−0.46	2.2E-04
203153_at	IFIT1	interferon-induced protein with tetratricopeptide repeats 1	−0.46	2.9E-04
211968_s_at	HSP90AA1	heat shock protein 90 kDa alpha (cytosolic), class A member 1	−0.45	5.6E-06
226800_at	EFCAB7	EF-hand calcium binding domain 7	−0.45	9.2E-09
225312_at	COMMD6	COMM domain containing 6	−0.44	6.1E-03
201699_at	PSMC6	proteasome (prosome, macropain) 26S subunit, ATPase, 6	−0.44	4.4E-07
222848_at	CENPK	centromere protein K	−0.44	2.4E-05
212587_s_at	PTPRC	protein tyrosine phosphatase, receptor type, C	−0.43	1.7E-04
219239_s_at	ZNF654	zinc finger protein 654	−0.43	3.0E-07
205849_s_at	UQCRB	ubiquinol-cytochrome c reductase binding protein	−0.43	2.7E-03
214453_s_at	IFI44	interferon-induced protein 44	−0.43	6.8E-05
227152_at	C12orf35	chromosome 12 open reading frame 35	−0.43	7.2E-05
200061_s_at	RPS24	ribosomal protein S24	−0.42	5.8E-03
205809_s_at	WASL	Wiskott-Aldrich syndrome-like	−0.42	4.0E-05
222616_s_at	USP16	ubiquitin specific peptidase 16	−0.42	6.0E-07
219356_s_at	CHMP5	chromatin modifying protein 5	−0.42	2.4E-05
244042_x_at	NA	NA	−0.41	4.0E-05
205871_at	PLGLA	plasminogen-like A	−0.41	1.4E-06
235653_s_at	THAP6	THAP domain containing 6	−0.41	1.7E-06
219387_at	CCDC88A	coiled-coil domain containing 88A	−0.41	6.8E-05
202110_at	COX7B	cytochrome c oxidase subunit VIIb	−0.41	4.0E-03
209795_at	CD69	CD69 molecule	−0.41	4.5E-05
224786_at	SCOC	short coiled-coil protein	−0.40	2.4E-03
221728_x_at	XIST	X (inactive)-specific transcript (non-protein coding)	−0.40	3.2E-05
214218_s_at	XIST	X (inactive)-specific transcript (non-protein coding)	−0.40	3.9E-04
212391_x_at	RPS3A	ribosomal protein S3A	−0.40	3.6E-04
202411_at	IFI27	interferon, alpha-inducible protein 27	0.41	6.5E-03
228582_x_at	MALAT1	Metastasis associated lung adenocarcinoma transcript 1 (non-protein coding)	0.49	5.8E-03

*NA* Not Available

^a^fold change (Log_2_ ratio)

These 45 genes then were input to the MetaCore reaction pathways analysis. The data indicated that Ph-positive allergic rhinitis patients who received acupoint herbal plaster applications significantly induced several pathways (*p* < 0.01; Table 10). Among the 45 down-regulated genes, pathway analysis identified significant involvement of the “Oxidative phosphorylation pathway” (*p* < 0.0001). Network analysis also identified “Protein folding_Response to unfolded proteins,” “Immune response Antigen presentation,” and “Immune response Phagosome in antigen presentation” as significant (*p* < 0.001) relative to the 45 down-regulated genes.

**Table 10:** Metacore process map for the 45 genes that were down-regulated in Ph-positive patients with allergic rhinitis following acupoint herbal paste treatment

Process map of down-regulated genes in Ph(+)			
Maps	*P* value	Filter Genes^a^	Map genes^b^
Protein folding_Response to unfolded proteins	2.3E-04	2 (HSP90AA1, HSP90B1)	69
Immune response_Antigen presentation	3.3E-04	3 (PTPRC, HSP90AA1, HSP90B1)	197
Immune response_Phagosome in antigen presentation	7.4E-04	3 (WASL, HSP90AA1, HSP90B1)	243

^a^Number of filter genes in the map

^b^Number of genes in the map

## Discussion

Allergic rhinitis likely results from an imbalance in the Th1 and Th2 cell-mediated inflammatory responses [20, 21]. In addition to the hygiene hypothesis causing deviation of the Th1 and Th2 balance and reduced immune suppression, investigators have implicated decreases in T-regulatory (Treg) activity in allergy diseases [42, 43]. People suffering from allergies, usually have a reduced Th1 reaction and a predominant Th2 response. Th1 cells tended to decrease in patients with allergic rhinitis, whereas Th2 cells were significantly increased. Significant deviations from the normal Th1/Th2 ratio may be associated with the incidence of allergic diseases [18, 20, 44]. A study examining allergic inflammation that focused on Th2 cytokines (IL-4, IL-5, IL-9, and IL-13) reported that these cytokines recruited cells that induced allergic inflammation via chemokine secretion [44]. Few reports have described human allergic inflammation with respect to cytokine antagonists [19, 21, 45]. Although strengthening the Th1 response is regarded as a novel therapeutic approach for allergic rhinitis, this method has not been applied clinically [19, 21]. A restructuring of the Th1 and Th2 responses in patients with allergic rhinitis may be accomplished with acupuncture [16, 17]. Studies have shown that acupuncture treatment of allergic inflammation can maintain the equilibrium between Th1 and Th2 cells and between Tregs and Th2 cells [16, 17].

Many patients choose acupoint herbal plaster treatments for allergic rhinitis in Taiwan [3–5] and mainland China [6, 7]. We previously examined the efficacy of acupoint herbal plaster treatment for allergic rhinitis [13]. The present study is the first to apply the RQLQ to comprehensively assess the effects of acupoint herbal plaster on allergic rhinitis symptoms. Our results suggest that acupoint herbal plaster is a safe, effective, and convenient treatment for allergic rhinitis. A comparison of baseline characteristics before treatment between Ph-positive and Ph-negative patients showed no differences, with the exception of total IgE levels (Table 1). The RQLQ results after the fourth treatment of 19 Ph-positive patients indicated symptom improvements in six of seven categories (activity, non-hay fever symptoms, practical problems, nasal symptoms, eye symptoms, emotional symptoms; Tables 2 and 3). In contrast, the four Ph-negative volunteers (−) reported symptom improvements in only two categories (nasal symptoms, emotional symptoms; Tables 2 and 3). These results are similar to those found in our previous report on acupuncture treatment for allergic rhinitis [16, 17]; however, the herbal plaster treatment was noninvasive and easy to apply. The degree of symptom improvement among Ph-positive allergic rhinitis patients was different with the Ph-negative group, indicating that the acupoint herbal plaster treatment in these patient groups evoked distinct physiological responses. Due to its preliminary nature, this study has some limitations including the lack of a control group or a safety assessment.

In this study, the average total serum IgE levels tended to increase in Ph-positive and -negative groups following the fourth herbal plaster treatment, but the changes were not statistically significant (Table 4 and 5). This result is similar to that of our previous acupuncture study [16, 17] and may indicate that reducing total IgE synthesis is not the primary mechanism of acupoint herbal plaster treatment of allergic rhinitis.

The Ph-positive and -negative groups exhibited different gene expression trends after acupoint herbal plaster treatment (Fig. 2 and Table 7). This supports the results of the RQLQ, and indicates that the patient groups respond differently to acupoint herbal plaster.

Pathway analysis of the differentially expressed genes indicated that “Immune response_IL-13 signaling via JAK-STAT” and “Inflammation_Interferon signaling” pathways corresponded to down- and up-regulated genes, respectively, between Ph-positive and Ph-negative patients (Fig. 2b and Table 8). Since a Th1/Th2 cytokine imbalance contributes to the etiology and pathogenesis of allergic rhinitis, understanding the mechanisms of this disease will help to find novel targets for therapy. Th1 cells secrete primarily IL-2, IFNγ, IL-3, and GM-CSF, whereas Th2 cells secrete IL-3, IL-4, IL-5, IL-10, IL-13, and GM-CSF [22]. Cytokines released after activation of T-cell receptors interact with cytokine receptors on mononuclear cells and activate these cells via the JAK-STAT (Janus kinase and signal transducers and activators of transcription) pathway. The JAK-STAT pathway is involved in histamine-mediated regulation of the Th2 cytokines IL-5, IL-10, and IL-13, and of the Th1 cytokine IFNγ [22]. IL-13 plays a central role in the promotion of an allergic inflammatory eosinophilic reaction in allergic diseases via IgE isotype switching. IFNγ down-regulates the secretion of certain Th2 cytokines [22]. Local administration of IFNγ in mice prevented antigen-induced eosinophil infiltration into the trachea and normalized airway function. However, recombinant subcutaneous administration of IFNγ had no benefit in the treatment of steroid-dependent asthma [22]. Pathways that downregulated IL-13 signaling via JAK-STAT and upregulated Interferon signaling pathways were differentially expressed between Ph-positive and Ph-negative patients with allergic rhinitis after acupoint herbal paste treatment; however, further studies are necessary to confirm these results.

Several pathways were significantly induced in Ph-positive allergic rhinitis patients who received acupoint herbal plaster applications. Phagosomal immune response in antigen presentation was noted due to an immune response to the herbal plater treatment (Table 10). Macrophages function to clear infectious particles, and this process involves engulfing microbes into phagosomes where they are lysed and degraded. Phagosomes are pivotal in linking both the innate and adaptive immune responses [46]. Phagosomal proteins regulated by IFNγ include proteins expected to alter phagosome maturation, enhance microbe degradation, trigger the macrophage immune response, and promote antigen loading on major histocompatibility complex (MHC) class I molecules [46]. IFNγ delays phagosomal acquisition of lysosomal hydrolases and peptidases to aid in antigen presentation, which is dependent on phagosomal networks of the actin cytoskeleton and vesicle-trafficking proteins, as well as Src kinases and calpain proteases [47].

In this preliminary study, Ph-positive patients with allergic rhinitis who received acupoint herbal plaster treatments manifested gene expression changes involved in the “Immune response_IL-13 signaling via JAK-STAT” pathway. These patients reported improved clinical symptoms of allergic rhinitis according to the RQLQ scale. Pathway analysis suggested that allergic rhinitis patients treated with acupoint herbal plaster improved their balance of Th1-derived pro-inflammatory cytokines versus Th2-derived anti-inflammatory cytokines. Our results indicate that acupoint herbal plaster application diminished allergic inflammation by maintaining an appropriate equilibrium between Th1 and Th2 cells.

## Conclusions

RQLQ and gene expression profiles indicated that patients with Ph-positive and -negative allergic rhinitis exhibit distinct physiological responses after receiving acupoint herbal plaster treatments. Gene expression levels were compared before and after acupoint herbal plaster application and in Ph-positive versus Ph-negative participants. In this preliminary study, we find that the IL-13 immune response via JAK-STAT signaling and interferon inflammation signaling were down- and upregulated, respectively, in the Ph-positive group. Further studies are required to verify these pathways in Ph-positive patients, and to determine the mechanism of such pathway dysregulation.
